# Building Capacity for Meaningful and Sustainable Exercise Research Partnerships With Community Organizations

**DOI:** 10.1093/geroni/igaf122.2115

**Published:** 2025-12-31

**Authors:** Laura Baehr, Megan George, Janet Bettger, Michael Wolf

**Affiliations:** Drexel University, Philadelphia, Pennsylvania, United States; Drexel University, Philadelphia, Pennsylvania, United States; The University of North Carolina at Chapel Hill, Chapel Hill, North Carolina, United States; Northwestern University, Evanston, Illinois, United States

## Abstract

Older adults should engage in lifestyle physical activity (aerobic, strength, and balance tasks) to maintain health, manage chronic conditions, and reduce frailty, yet only 11% achieve minimal weekly recommendations. Older adults with social considerations such as mobility limitations and/or socioeconomic disadvantage are at highest risk for physical inactivity and subsequent poor health outcomes compared to age matched peers. There is strong evidence that community-based exercise programs promote physical activity in older adults, yet the needs and perspectives of those most in need of these interventions aren’t prioritized during development, assessment, and implementation. The goal of this capacity building project is to employ the Explore, Prepare, Implement, Sustain (EPIS) Framework to the systematic assessment of community-based organizations (CBOs) providing free or low cost health promotion programs in Philadelphia, home to some of the most socioeconomically disadvantaged Americans with poverty rates nearly four times higher than suburban counties. To achieve this goal, we first explored CBOs to determine appropriateness for research collaborations by creating an exhaustive list through multiple inputs. The team leveraged known partnerships through local universities as well as web-based searches. A list of key search words was generated to capture CBOs, health promotion, and older adults. Repeated web-based searches inclusive of public databases were performed to collect CBO information, until a final list of potential CBOs (n = 217) was identified. The next step (ongoing) applies the Preparation phase of the EPIS Framework to interview all identified CBOs to determine goals, and interest in partnership to prepare for future collaborations.

